# Supplementary material for: Purification and Characterization of the Enzyme Fucoidanase from Cobetia amphilecti Utilizing Fucoidan from Undaria pinnatifida
Source: Foods. 2023 Apr 6;12(7):1555. doi: 10.3390/foods12071555 (PMC10094035; doi:10.3390/foods12071555)
Supplement: Supplementary file 1 [file foods-12-01555-s001.zip › foods-2227911-supplementary.pdf]

1. SDS-PAGE

1.1 The method of SDS-PAGE

The SDS-PAGE was determined to identify the purity and estimate the MW of fucoidanase as described by the method as Chen [50] reported.

1.2 The results of SDS-PAGE

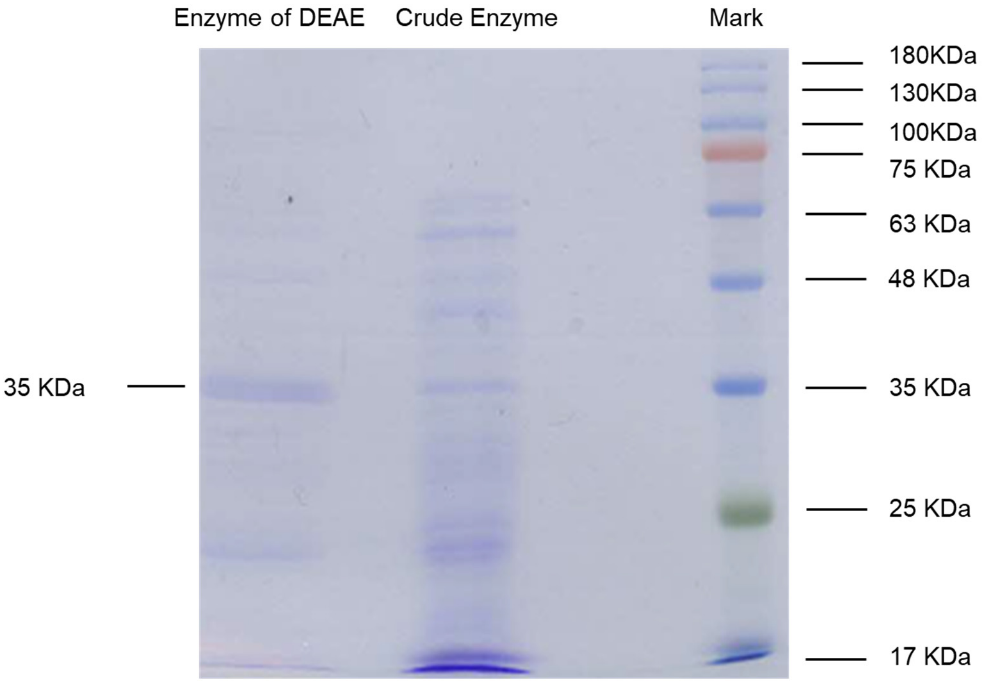

Figure S1. The SDS-PAGE results of fucoidanase.

The results showed that the fucoidanase mainly showed a single band on SDS-PAGE after purification by DEAE Sepharose Fast Flow column chromatography. According to the molecular weight corresponding to the mark, the molecular weight of the enzyme was preliminarily obtained as 35.0KDa.

2. Orthogonal Experiments

2.1 The method of Orthogonal Experiments

Orthogonal experiments with four factors and three levels (Table 1) were set up based on single factorial experiments with the interval time durations fixed at 4s.

Table S1. Factors of orthogonal experiment.

Level No.	Factors			
	A Ultrasonic Power /W	B Total Time /min	C Concentration of Bacterial / mg/mL	D Single Time /s
1	240	6	40	3
2	300	9	50	5
3	360	12	60	7

2.2 The results of Orthogonal Experiments

Table S2. The results of L9 (3^4) orthogonal experiment.

No.	Factors				Enzyme Activity/U
	A	B	C	D	
1	1	1	1	1	37
2	1	2	2	2	86.69
3	1	3	3	3	141.13
4	2	1	2	3	169.89
5	2	2	3	1	187.01
6	2	3	1	2	275.09
7	3	1	3	2	112.21
8	3	2	1	3	240.17
9	3	3	2	1	226.91
K1	7944.6	9573	16567.8	13527.6	—
k2	18959.7	15416.1	14504.7	14219.7	—
k3	17378.7	19293.9	13210.5	16535.7	—
R	11015.1	9720.9	3357.3	3008.1	—

The influence order of experimental factors on enzyme activity was as follows: A > B > C > D, namely ultrasonic power > total time > concentration of bacterial > single time. Based on the results (Table 2), the actual optimal group was $A_2B_3C_1D_2$ （No.6） and the theoretical optimal group was $A_2B_3C_1D_3$.

Table S3. Verification test results of enzyme activity in two groups.

No.	Factors				Enzyme Activity/U
	A	B	C	D	
actual optimal group	2	3	1	2	238.01
theoretical optimal group	2	3	1	3	231.87

According to the results (Table 3), there was little difference in enzyme activity between the two groups, the enzyme activity of actual optimal group was slightly higher than the theoretical optimal group. After the verification experiment, the ultrasonic power for optimal extraction state was 300 W, the concentration of bacterial was 40 mg/mL, the total time was 12min, and the single were 5 s, and the interval time durations was 4 s. The highest enzyme activity was reached at 275 U under the optimal reaction conditions.
